# Drug Use in Pregnancy; a Point to Ponder!

**DOI:** 10.4103/0250-474X.51941

**Published:** 2009

**Authors:** Punam Sachdeva, B. G. Patel, B. K. Patel

**Affiliations:** Department of Pharmacology, A. R. College of Pharmacy, Vallabh Vidyanagar-388 120, India; 1 Department of Pharmacology, Sat Kaival College of Pharmacy, Sarsa-388 365, India

**Keywords:** Teratogenic drugs, physiology of pregnancy, FDA categories of drugs, drug use in pregnancy

## Abstract

Pregnancy is a special physiological condition where drug treatment presents a special concern because the physiology of pregnancy affects the pharmacokinetics of medications used and certain medications can reach the fetus and cause harm. Total avoidance of pharmacological treatment in pregnancy is not possible and may be dangerous because some women enter pregnancy with medical conditions that require ongoing and episodic treatment (e.g. asthma, epilepsy, hypertension). Also during pregnancy new medical problems can develop and old ones can be exacerbated (e.g. migraine, headache) requiring pharmacological therapy. The fact that certain drugs given during pregnancy may prove harmful to the unborn child is one of the classical problems in medical treatment. In 1960's pregnant ladies who ingested thalidomide gave birth to children with phocomalia. Various other examples of teratogenic effects of drugs are known. It has been documented that congenital abnormalities caused by human teratogenic drugs account for less than 1% of total congenital abnormalities. Hence in 1979, Food and Drug Administration developed a system that determines the teratogenic risk of drugs by considering the quality of data from animal and human studies. FDA classifies various drugs used in pregnancy into five categories, categories A, B, C, D and X. Category A is considered the safest category and category X is absolutely contraindicated in pregnancy. This provides therapeutic guidance for the clinician. This article focuses on various aspects relating to drug use during pregnancy.

In pregnancy drug treatment presents a special concern due to the threat of potential teratogenic effects of the drug and physiologic adjustments in the mother in response to the pregnancy[1]. The physiology of pregnancy affects the pharmacokinetics of medications used and certain medications can reach the fetus and cause harm[2]. The concern about medication use during pregnancy and lactation has been influenced by historical events, including thalidomide crisis in the 1960's and the teratogenic effects discovered related to the use of diethylstilboestrol in 1971[3]. These events led the US Food and Drug Administration to establish strict regulations regarding drug labeling, the use of medications in pregnancy, requiring demonstrations of safety and efficacy of any drug before it becomes commercially available[4].

## PHYSIOLOGICAL CHANGES IN PREGNANCY

Pregnancy occurs when a sperm penetrates an egg. This is called fertilization and usually takes place in the woman's fallopian tube. The fertilized egg immediately begins to divide into a growing cluster of cells. Between 5-7 days after ovulation the fertilized egg implants into the wall of uterus and starts forming the placenta. The placenta maintains and nourishes the baby by enabling the transfer of O_2_, CO_2_, amino acids, fats, vitamins and minerals from the mother's blood. It also allows transfer of waste substances from the growing baby. From the time of implantation into the wall of uterus until approximately eighth week of life the baby is known as embryo. Development is rapid during this stage as the specialized cells begin to form the vital organs, nervous system, bones, muscles and blood. After the eighth week of pregnancy the developing baby is called a fetus. It is 2.4 cm long with most of internal organs formed and external features such as eyes, nose, mouth and ears start to appear[5].

As the fetus and placenta grow and place increasing demand on the mother, phenomenal alterations in metabolism occur. The most obvious physical changes are weight gain and altered body shape. Weight gain is due to increase in breast tissue, blood and water volume in the form of extra vascular and extra cellular fluid. Deposition of fat and protein and increased cellular water are added to maternal stores. The average weight gain during pregnancy is 12.5 kg. During normal pregnancy 1 kg weight gain is due to protein. Also plasma albumin levels are decreased and fibrinogen levels are increased. Total body fat increases during pregnancy. During second half of pregnancy plasma lipids increase but triglycerides, cholesterol and lipoproteins decrease soon after delivery. The ratio of LDL to HDL increases during pregnancy[6].

How a drug affects the fetus depends on the fetus's stage of development and the strength and dose of the drug[7]. Limited information exists regarding the effects of drugs in the period of conception and implantation. It is suggested that women who are at the risk of conceiving or who wish to become pregnant should withdraw all unnecessary medications 3-6 months before conception[8]. Certain drugs taken early in pregnancy (15-21 days after fertilization) during the period of blastogenesis may act in an all or nothing fashion; killing the foetus or not affecting it at all. During this early stage the fetus is highly resistant to birth defects. The fetus is highly vulnerable to birth defects between 3^rd^ week and 8th week after fertilization; which is the period of organogenesis. All major organs start developing during this period. Drugs reaching the fetus during this stage may cause a miscarriage, an obvious birth defect, or a permanent but subtle defect, that is noticed later in life. At 9^th^ week the embryo is referred to as a fetus. Development during this time is primarily maturation and growth. Exposure to drugs during this period is not associated with major congenital malformations but they may alter the growth and function of normally formed organs and tissues[7].

The effect of a medication also depends on the dose that reaches the fetus. This dose is affected by the maternal dose, distribution of the drug in maternal blood stream, placental function, maternal and fetal genetic and physiologic status as well as exposure to other drugs, chemicals or environmental hazards[9].

## PHARMACOKINETICS IN PREGNANCY

The unique physiologic changes of pregnancy affect the pharmacokinetics of medications used by pregnant women. During pregnancy a woman's plasma volume increases by 30-50% and cardiac output and glomerular filtration rate also increase in similar proportion. These factors contribute to lower circulating concentration of some drugs (especially those excreted by kidney) in a pregnant woman and possibly to subtherapeutic drug levels. Also there is increase in body fat during pregnancy; which increases the volume of distribution of fat soluble drugs. A decrease in plasma albumin concentration during pregnancy increases the volume of distribution for highly protein bound drugs e.g. anticonvulsants. But the unbound drugs are excreted out more rapidly by the kidney and liver; and this offsets the effect of increased volume of distribution. Due to the effect of progesterone, gastric emptying time is decreased particularly in the third trimester thus delaying the onset of effect of the drug[9].

Concurrent use of other common medications during pregnancy such as antacids, iron and vitamins could also bind and inactivate some drugs. Intramuscular absorption of drug is generally more rapid due to increased blood flow; which enhances systemic drug absorption and the rate of onset of action[9]. Lastly estrogen and progesterone alter hepatic enzyme activity; which can increase drug accumulation or decrease elimination of some drugs[10].

## PLACENTAL TRANSFER OF DRUGS

The placenta; the product of conception is the functional unit between fetal blood and maternal blood. The functions of the placenta include nutrition, respiration, metabolism, excretion and endocrine activity to maintain fetal and maternal well-being. In order for a drug to cause a teratogenic or pharmacological effect on the fetus, it must cross from maternal circulation to fetal circulation through the placenta by diffusion[8]. The rate of transfer depends on the chemical properties of the drug such as protein binding, pH difference, lipid solubility and molecular weight of the drug[11]. Only free unbound drug crosses the placenta. During pregnancy maternal plasma albumin decreases while fetal albumin increases. As a result the concentration of free drug increases which crosses the placenta to reach the fetus. Fetal pH is slightly more acidic than maternal pH and so weak bases are more likely to cross the placenta[12]. Moderately lipid soluble drugs can easily diffuse across the placental membrane. Drugs with low molecular weight (<500 g/mol) diffuse freely across the placenta. Drugs with a higher molecular weight (between 500-1000 g/mol) cross the placenta less easily, while a few drugs with a high molecular weight (>1000 g/mol) do not cross the placental membrane[11]. Transplacental transfer of drugs increases in the third trimester due to increased maternal and placental blood flow, decreased thickness and increased surface area of the placenta[9].

## PREGNANCY AND DRUG USE

Drugs play an important role in improving human health and promoting well-being. However to produce the desired effect, they have to be safe, efficacious and have to be used rationally[13]. In general, drugs unless absolutely necessary should not be used during pregnancy because drugs taken by a pregnant woman can reach the fetus and harm it by crossing the placenta, the same route taken by oxygen and nutrients, which are needed for the growth and development of fetus[7].

While avoiding medications when pregnant may be desirable, it is often not possible and may be dangerous because some women enter pregnancy with medical conditions that require ongoing and episodic treatment (e.g. asthma, epilepsy, hypertension). Also during pregnancy new medical problems can develop and old ones can be exacerbated (e.g. migraine headache) requiring pharmacological therapy. Failure to manage conditions like these may affect the health of both the mother and her infant[14]. Also some drugs like vitamins, minerals, iron and dietary supplements are essential for the health of pregnant woman and the fetus. It has been reported that about 8% of pregnant women need drug treatment due to various chronic diseases and pregnancy related complications[13]. Many women take medications in the early weeks of pregnancy before realizing that they are pregnant. About 59% of pregnant women are prescribed a medication other than a vitamin or mineral supplement. About 13% of pregnant women take a dietary herbal supplement[14]. More than 90% of pregnant women take prescription or nonprescription (over-the-counter) drugs or use social drugs such as tobacco or alcohol or illicit drugs at sometime during pregnancy[7]. The fact that certain drugs given during pregnancy may prove harmful to the unborn child is one of the classical problems in medical treatment[15].

Pregnant women are usually excluded from medical trials and results from animal studies need not apply to human population. Hence treating pregnant women with some drugs is a problem and most clinicians have a rather restricted approach to the use of drugs during pregnancy. Fear of causing fetal harm and death through medication use in pregnancy has resulted in many challenges to clinical research about the safety of drugs in pregnancy. Therefore medication safety information in pregnancy is actually obtained through case reports, epidemiological studies and animal studies; all of which have limitations, that make determining risks of a drug use during pregnancy difficult[4].

A study in 2001 found that there was not enough information about the risk or safety of more than 90% medications approved by FDA between 1980 and 2000 when taken during pregnancy. This makes it difficult for women and health care providers to decide whether to use medications during pregnancy or not[14].

Despite the paucity of information on the safety of drugs in pregnancy, the statistics on over the counter (OTC) and prescription drugs used in pregnancy indicate that drug use in pregnancy is wide spread[15]. About 2-3% of all birth defects result from use of drugs. However drugs are sometimes essential for the health of pregnant women and fetus. A health care practitioner may recommend that women take certain vitamins and minerals during pregnancy[7]. Drugs are also used for treatment of some common symptoms associated with pregnancy such as aches and pains, nausea and vomiting, and edema[18]. Medications may also be prescribed to treat conditions occurring during but unrelated to pregnancy such as upper respiratory infections, urinary tract infections and gastrointestinal upsets to name some. Also pregnant woman may be using medications to treat pre existing chronic conditions such as epilepsy, hypertension or psychiatric disorders or to treat pregnancy related disorders such as pregnancy induced hypertension, to induce labor or to facilitate lung maturity in the fetus expected to be delivered preterm[16]. Also this patient population may be exposed to any other agents that may have an adverse effect on fetus[8]. It therefore becomes important to examine the pattern of drug use in pregnancy to see to what extent there may be room for improvement in the light of current knowledge[17].

## HOW DRUGS AFFECT THE FETUS[7]

Drugs that a pregnant woman takes can affect the fetus in several ways. They can act directly on the fetus causing damage or abnormal development leading to birth defects or death. They can also alter the function of the placenta usually by constricting blood vessels and reducing the blood supply of oxygen and nutrients to the fetus from the mother and thus resulting in a baby that is underweight and underdeveloped. Moreover they can cause the muscles of the uterus to contract forcefully; indirectly injuring the fetus by reducing the blood supply or triggering pre-term labor and delivery.

## FDA CATEGORIES FOR DRUG USE IN PREGNANCY[18]

In 1979, the Food and Drug Administration developed a system determining the teratogenic risk of drugs by considering the quality of data from animal and human studies. It provides therapeutic guidance for the clinician. Category A is considered the safest category but some drugs from categories B, C and D are also used during pregnancy. Category X is the only rating that denotes a drug is absolutely contraindicated for use during pregnancy (Table 1). Some of the drugs commonly used during pregnancy and their categories (as per FDA categorization) are mentioned in the table given below (Table 2). Some of the drugs have been proved to be harmful to the fetus and so their use during pregnancy is contraindicated. (Table 3)

**TABLE 1:** FDA CATEGORIZATION OF DRUGS FOR USE IN PREGNANCY[18]

Category	Description
A	Adequate, well-controlled studies in pregnant women have not shown an increased risk of fetal abnormalities.
B	Animal studies have revealed no evidence of harm to the fetus; however, there are no adequate and wellcontrolled studies in pregnant women. Or
	Animal studies have shown an adverse effect, but adequate and well-controlled studies in pregnant women have failed to demonstrate a risk to the fetus.
C	Animal studies have shown an adverse effect and there are no adequate and well-controlled studies in pregnant women. Or
	No animal studies have been conducted and there are no adequate and well-controlled studies in pregnant women.
D	Studies, adequate well-controlled or observational, in pregnant women have demonstrated a risk to the fetus. However, the benefits of therapy may outweigh the potential risk.
X	Studies, adequate well-controlled or observational, in animals or pregnant women have demonstrated positive evidence of fetal abnormalities. The use of the product is contraindicated in women who are or may become pregnant.

FDA categorization of drugs for use in pregnancy

**TABLE 2:** COMMONLY USED DRUGS IN PREGNANCY AND THEIR CATEGORIES[19]

Drugs	Category
Analgesics and Antipyretics	B and C
Acetaminophen	B
Phenacetin	B
Aspirin	C
Antiemetics	B and C
Doxylamine	B
Meclizine	B
Cyclizine	B
Dimenhydrinate	B
Antibiotics	B, C and D
Penicillin, Ampicillin, Amoxycillin,	B
Cloxacillin Cephalosporins	B
Erythromycin	B
Gentamicin	C
Amikacin	C/D
Streptomycin	D
Sulphonamides	B/D
Tetracyclines	D
Amoebicides	B
Metronidazole	
Anthelmentics	B
Piperazine	
Mebendazole	
Antimalarials	C
Antifungals	C
Anti TB Drugs	B and C
Ethambutol	B
INH	C
Rifampicin	C
Pyrazinamide	C
PAS	C
Vitamins	
B,C,D,E,folic acid	A
Hormones	
Thyroxin	A
Androgens	X
Estrogens	X
Progestogens-	
Hydroxyprogestrone	D
Medroxyprogestrone	D
Norethindrone	X
Norgestrel	X
Bronchodilators	C

List of some of the commonly used drugs during pregnancy along with their categories as per FDA categorization

**TABLE 3:** MEDICATIONS CONTRAINDICATED IN PREGNANCY

Drug	Comments
Vitamin A and its derivatives including isotretinein, accutane and etretinate.	Significant risk of spontaneous abortion[20] and risk of many significant anomalies[21]
ACE inhibitors	May cause kidney damage in the fetus when used in II and III trimester, decrease in the amount of amniotic fluid and deformities of face, limbs and lungs[7]
Anticoagulants- warfarin	Use during I trimester produces defects like nasal hypoplasia and a depressed nasal bridge; termed as Fetal warfarin Syndrome. Use during II and III trimesters is associated with increased risk of fetal malformations[8].
- Heparin	Safe but if taken for long time osteoporosis and decrease in number of platelets in pregnant women occurs[7].
Estrogen and Androgens	Genital tract malformations[8].
Thyroid preparations-	
Methimazole	Overactive and enlarged Thyroid gland
Carbimazole	Overactive and enlarged Thyroid gland
Radioactive iodine	Underactive Thyroid gland in fetus
Propylthiouracil	Safe[7].
Anticonvulsants-	
Carbamazepine	Risk of birth defects
Phenytoin, Phenobarbitone	Bleeding problem in the newborn which can be prevented if pregnant woman takes Vit. K by mouth every day for a month before delivery or if the newborn baby is given an injection of Vit. K soon after birth[7].
	Risk of birth defects.
Trimethadione	Increased risk of miscarriage in the women
Sodium valproate	Increased risk of birth defects in fetus; including a cleft palate and abnormalities of the heart, face, skull, hands or abdominal organs[7].
Antidepressants- Lithium	Birth defects (mainly of the heart), lethargy, decreased muscle tone, underactivity of Thyroid gland and nephrogenic diabetes insipidus in the new born. Ebstein's anomaly (tricuspid valve malformation) has been reported in a number of foetuses exposed to this drug[7].
NSAIDs	
Aspirin and other Salicylates	Delay in start of labor, premature closing of ductus arteriosus, jaundice, brain damage in the fetus and bleeding problems in the woman during and after delivery and in the newborn[7].
Antibiotics- Tetracycline	Slowed bone growth, permanent yellowing of the teeth and increased susceptibility to cavities in the body[7].
Chloramphenicol	Gray Baby Syndrome[7].
Ciprofloxacin	Possibility of joint abnormalities (seen in animals)[7]
Kanamycin and Streptomycin	Damage to fetus's ear resulting in deafness (risk of ototoxicity)[7]
Sulfonamides	Jaundice and brain damage in newborn[7]
Antineoplastic agents-	
Busulfan	Birth defects such as less than expected growth before birth, underdevelopment of lower jaw, cleft palate, abnormal development of skull bones, spinal defects, ear defects and club foot[7].
Chlorambucil	
Cyclophosphamide	
Methotrexate	
Oral Hypoglycemic drugs	A very low level of sugar in the blood of newborn. Inadequate control of diabetes in the pregnant woman[7]
Chlorpropamide	
Tolbutamide	

List of some of the drugs whose use is contraindicated during pregnancy along with the harmful /damaging effects they may produce on the fetus

## SOCIAL DRUGS

In addition to counseling pregnant women regarding use of various prescribed and non-prescribed medications during pregnancy, other substances that are also used by some women during pregnancy should not be overlooked. They should be informed about the risk of using the following substances during pregnancy[18].

### Cigarette smoking:

Maternal smoking is one of the few known preventable causes of prenatal morbidity and mortality[14]. The most consistent effect of smoking on the fetus during pregnancy is a reduction in birth weight. Birth defects of heart, brain and face are also more common among babies of smokers. Risk of sudden infant death syndrome (SIDS), mis-located placenta (placenta previa), premature detachment of placenta (placenta abruptio), premature rupture of the membranes, preterm labor, uterine infections, miscarriages, stillbirths, premature births are increased[7]. Changes in uterine and placental oxygenation may be the cause of infant death, pre-maturity or spontaneous abortions. Therefore all women should be informed of the risk of smoking on the fetus and encouraged to quit smoking during pregnancy[18].

### Alcohol:

Foetal Alcohol Syndrome is one of the most serious consequences of drinking during pregnancy[7]. Worldwide incidence of Fetal Alcohol Syndrome is 1:2000 live births[18]. Risk of miscarriage almost doubles for women who drink alcohol in any form during pregnancy and birth weight of babies is substantially below normal. This syndrome includes inadequate growth before or after birth, facial defects, a small head, mental retardation and abnormal behavioral development[7]. Factors that contribute to the expression of this syndrome are poor nutrition, smoking, drug abuse, genetic disposition and low socio-economic status[18].

### Caffeine:

Caffeine is found in various quantities in many beverages, analgesics, diet aids and stimulants, Hence it is the most commonly ingested drug during pregnancy[18]. Evidence suggests that consuming caffeine during pregnancy poses little or no risk to the fetus. Caffeine contained in coffee, tea, some sodas, chocolates and some drugs is a stimulant that readily crosses the placenta to the fetus[7]. If taken in high dose it may stimulate the fetus increasing heart and breathing rate[18]. Caffeine also may decrease blood flow across placenta and decrease the absorption of iron; increasing risk of anemia[7].

### Illicit drugs:

Use of illicit drugs like cocaine and opioids during pregnancy can cause complications and serious problems in the developing fetus and the newborn. Growth of fetus is likely to be inadequate and premature birth defects are more common. Cocaine crosses the placenta, constricts the blood vessels reducing blood flow to the fetus. The reduced blood and oxygen supply to the fetus slows the growth of bones and intestine. Use of cocaine can also cause complications during pregnancy. Among women who use cocaine throughout pregnancy, 31% have preterm delivery and 15% have premature detachment of placenta. The chances of miscarriage also increase[7].

## CONCERNS WITH OTC DRUGS

In India due to easy availability of drugs coupled with inadequate health services, increased proportion of drugs are used as self medications for common complaints and infective conditions as compared to prescribed drugs. Hence these consumers always face the threat of adverse drugs reactions and drug interactions[13]. While many OTC drugs can be used during pregnancy under a physician's supervision, some are known to be unsafe. As indicated on product labels, women who are pregnant, who may be pregnant, or who are nursing should consult a doctor before taking OTC medication[22].

Aspirin is one OTC drug that should be avoided in the last three months of pregnancy. In 1990, the FDA issued a warning that it is especially important not to use aspirin during the last trimester of pregnancy unless specifically directed to do so by a physician because it may cause problems in the unborn child or complications during delivery. OTC non-steroidal antiinflammatory drugs such as ibuprofen also carry the same warning about use during the third trimester[22].

Experts stress that much is not known about the effects of herbs and dietary supplements on a growing fetus to determine whether they are safe to use during pregnancy. Hence it should not be assumed that a product is safe for use during pregnancy because it is sold over-the-counter and is labeled as natural[22].

## CONCLUSIONS

The unique nature of physiology of pregnancy presents challenges for pharmaceutical treatment of chronic and acute disorders and for symptom management of many complaints associated with pregnancy. It is the responsibility of all clinicians including pharmacists to counsel patients with complete, accurate and current information on the risks and benefits of using medications during pregnancy[18]. Counseling women who have had exposure to drugs about risk of teratogens involves accurately identifying exposure and quantifying the magnitude of exposure. This may be straightforward for prescribed drugs but it can be much more difficult with ethanol or other illicit substances or OTC drugs[23]. Also when selecting drugs to be used in pregnancy effectively, drugs that have been in use for a long time are often preferable because fetal safety has been established even though newer alternatives may be available[24].

## References

[1] Banhidy F, Lowry RB, Czeizel AE (2005). Risk and benefit of drug use during pregnancy. Int J Med Sci.

[2] Deborah E, McCarter, Spaulding MS (2005). Medications in pregnancy and lactation. Amer J Maternal Child Nursing.

[3] Melton MW (1999). Take two Aspirin or not? Risk of medication use during pregnancy. Mother Baby J.

[4] Ward RW (2001). Difficulties in the study of adverse fetal and neonatal effects of drug therapy during pregnancy. Semin Perinatol.

[5] HON Foundation NHS Choices [Internet].

[6] Moore PJ, De Cherney AH, Pernoll ML (1994). Maternal Physiology during Pregnancy. Current obstetrics and gynaecological diagnosis and treatment.

[7] Porter RS (2004). The Merck Manual's Online Medical Library.

[8] Sorensan MK, Phillips BB, Mutnick AH, Shargel L, Mutnick A (2004). Drug use in specific patient populations: Pediatric, Pregnant, Geriatric. Comprehensive Pharmacy Review.

[9] Yankowitz J, Niebyl JR (2001). Drug therapy in pregnancy.

[10] Hansen W, Yankowitz J (2002). Pharmacologic therapy for medical disorders during pregnancy. Clin Obstet Gynaecol.

[11] Kraemer K (1997). Placental transfer of drugs. Neonatal Network.

[12] Loebstein R, Lalkin A, Koren G (1997). Pharmacokinetic changes during pregnancy and their clinical relevance. Clin Pharmacokinet.

[13] Sharma R, Kapoor B, Verma U (2006). Drug utilization pattern during pregnancy in North India. J Med Sci.

[14] Andrade SE, Gurwitz JH, Davis RL, Chan KA, Finkelstein JA, Fortman K (2004). Prescription drug use in pregnancy. Am J Obstet Gynaecol.

[15] Yaffe SJ (2002). Drugs in pregnancy and lactation.

[16] Splinter MY, Sagraves R (1997). Prenatal use of medications by women giving birth at a university hospital. South Med J.

[17] De Jong LT, Van den Berg PB (1990). A study of drug utilization during pregnancy in the light of known risks. Int J Risk Safety Med.

[18] Pangle BL, Herfindal ET, Gourley DR (2006). Drugs in Pregnancy and Lactation. Text book of Therapeutics, Drug and Disease Management.

[19] Nanavati MS (1994). Obstetrics Handbook for Maternal Health.

[20] Briggs GG (2002). Drug effects on the fetus and breastfed infants. Clin Obstet Gynaecol.

[21] Lewes L (2000). Which medications are safe in pregnancy?. Patient Care.

[22] Meadows M (2001). Pregnancy and the drug dilemma. FDA Consumer Magazine.

[23] Koren G, Pastuszak A (1998). Drugs in pregnancy. N Eng J Med.

[24] Koren G (1994). Maternal Fetal Toxicology.

